# Modulation of Venlafaxine Hydrochloride Release from Press Coated Matrix Tablet

**DOI:** 10.4103/0250-474X.42974

**Published:** 2008

**Authors:** M. C. Gohel, C. D. Soni, S. A. Nagori, K. G. Sarvaiya

**Affiliations:** Department of Pharmaceutics and Pharmaceutical Technology, L. M. College of Pharmacy, Navrangpura, Ahmedabad-380 009, India

**Keywords:** Venlafaxine hydrochloride, modified release, press coating, hydroxypropylmethylcellulose

## Abstract

The aim of present study was to prepare novel modified release press coated tablets of venlafaxine hydrochloride. Hydroxypropylmethylcellulose K4M and hydroxypropylmethylcellulose K100M were used as release modifier in core and coat, respectively. A 3^2^ full factorial design was adopted in the optimization study. The drug to polymer ratio in core and coat were chosen as independent variables. The drug release in the first hour and drug release rate between 1 and 12 h were chosen as dependent variables. The tablets were characterized for dimension analysis, crushing strength, friability and *in vitro* drug release. A check point batch, containing 1:2.6 and 1:5.4 drug to polymer in core and coat respectively, was prepared. The tablets of check point batch were subjected to *in vitro* drug release in dissolution media with pH 5, 7.2 and distilled water. The kinetics of drug release was best explained by Korsmeyer and Peppas model (anomalous non-Fickian diffusion). The systematic formulation approach enabled us to develop modified release venlafaxine hydrochloride tablets.

Venlafaxine hydrochloride, a novel antidepressant, differs structurally from other currently available antidepressants^1^. It is a white crystalline solid, freely soluble in water (534 mg/ml) and possesses serotonin/noradrenaline uptake inhibiting effect^2^–^6^. The dose of venlafaxine hydrochloride ranges from 75 to 350 mg per day. The steady state half life of venlafaxine and its active metabolite (o-desmethylvenlafaxine) is 5 and 11 h, respectively. The short half life of the drug indicates the need for modified release dosage form. Venlafaxine hydrochloride is currently available as immediate release tablet and as an extended release capsule under the brand names of Effexor^®^ (Wyeth Ayerst) and Effexor XR^®^ (Wyeth Ayerst). Modified release solid oral dosage forms of venlafaxine hydrochloride offer the advantages of improved patient compliance and decreased side effects^7^–^9^. Majority of oral sustained and controlled release drug delivery systems are based on either gel forming matrix or coated formulations, or the combination thereof^10^. Matrix tablet systems are most popular due to ease in scale-up^11^. Numbers of patents have been granted for modified release venlafaxine hydrochloride formulations. A European patent, assigned to Sherman *et al*., describes encapsulated extended release dosage form in the form of coated spheroids^7^. Sela disclosed preparation of sustained release compositions containing coated non pareil cores^12^. Molenda disclosed capsule formulation containing coated microgranules of venlafaxine^13^. A US patent describes an extended release composition of venlafaxine hydrochloride in matrix tablet dosage form, in which the drug is mixed with a combination of hydrophilic and hydrophobic matrix forming components^14^. Seth obtained sustained release of venlafaxine by coating drug containing core with a mixture of water soluble and semipermeable film forming polymer^15^. A Canadian patent assigned to Bhattacharya *et al*., describes zero order sustained release dosage forms^16^. US patent assigned to Vaya *et al*., describes dual retard technique to effectively control the release rate of venlafaxine^17^. The present research endeavour was directed towards the development of a modified release dosage form (12 h) of venlafaxine in the form of tablets without infringing the existing patents.

## MATERIALS AND METHODS

Venlafaxine hydrochloride and ethyl cellulose were received as gift samples from Torrent Pharmaceuticals (India). Hydroxypropylmethylcellulose K4M (HPMC K4M) and K100M (HPMC K100M) were received as gift from Zydus Cadila (India). Magnesium stearate was purchased from Laser Laboratories (India). Dicalcium phosphate dihydrate (DCPD) and acetone were purchased from Finar Chemicals Pvt. Ltd. (India). The other chemicals were of laboratory grade.

### Preliminary work:

The mixture of venlafaxine hydrochloride and HPMC K4M was wet massed using 5% w/v solution of ethyl cellulose in acetone. The wet mass was passed through 30# to obtain granules. The granules were dried at 60^º^ in a tray drier. The granules of 30/60# size were lubricated with 1% w/w magnesium stearate. The tablets with 10 kp crushing strength were prepared on a single station tablet press (Cadmach Machines Ltd., India). Table 1 depicts composition and results of evaluation of the preliminary batches (VH1 to VH3).

**TABLE 1:** COMPOSITION AND RESULTS OF VENLAFAXINE MATRIX TABLETS

Ingredients+	Batch code		
	
	VH1	VH2	VH3
Venlafaxine hydrochloride	37.5	37.5	37.5
HPMC K4M	37.5	75	112.5
Ethyl cellulose	7.5	7.5	7.5
Magnesium stearate Granules	1	1	1.5
Angle of repose (°) Tablet	34	38	38
Diameter (mm)	8.0±0.01	8.0±0.01	8.0±0.01
Thickness (mm)	2.0±0.03	2.3±0.03	2.7±0.03
Friability (%)	0.7	0.6	0.6

+ The quantity of raw material is expressed in mg per tablet.

### Preparation of venlafaxine hydrochloride press coated tablet:

Core tablets were prepared using venlafaxine hydrochloride, dicalcium phosphate dihydrate, HPMC K4M, ethyl cellulose and magnesium stearate. The procedure employed for the preparation of venlafaxine hydrochloride core tablet was identical to that described above (preliminary work). The core tablets were compressed at low pressure to produce tablets with 7 kp crushing strength to enable press coat to stick well with the core.

Press coat layer (57% weight gain) contained venlafaxine hydrochloride, dicalcium phosphate dihydrate, HPMC K100M, ethyl cellulose and magnesium stearate. Granules of venlafaxine hydrochloride were prepared by wet granulation technique. One-half of the required quantity of granules was transferred in the die. The core tablet was then carefully placed in the centre of the die and the remaining half of the granules was placed in the die. Press coated tablet with 13 kp crushing strength were prepared on single station tablet press (Cadmach Machines Ltd., India). Different batches were prepared by altering the ratio of venlafaxine hydrochloride to HPMC to modify the drug release (Table 2). A 3^2^ full factorial design was used for optimization. Table 3 shows the design layout for the optimization study and the responses. The press coated tablets were characterized for dimension analysis, friability and *in vitro* drug release.

**TABLE 2:** COMPOSITION OF VENLAFAXINE PRESS COATED TABLETS

Ingredients+	Batch code										
	
	VP0	VP1	VP2	VP3	VP4	VP5	VP6	VP7	VP8	VP9	VP10
Core:											
Venlafaxine HCl	37.5	30	30	30	30	30	30	30	30	30	30
HPMC K4M	93.7	30	30	30	60	60	60	90	90	90	78
DCPD*	9.8	81	81	81	51	51	51	21	21	21	33
Ethyl cellulose	7.5	7.5	7.5	7.5	7.5	7.5	7.5	7.5	7.5	7.5	7.5
Magnesium stearate	1.5	1.5	1.5	1.5	1.5	1.5	1.5	1.5	1.5	1.5	1.5
Coat:											
Venlafaxine HCl	-	7.5	7.5	7.5	7.5	7.5	7.5	7.5	7.5	7.5	7.5
HPMC K100M	56.2	18.7	37.5	56.2	18.7	37.5	56.2	18.7	37.5	56.2	40.8
DCPD*	23.6	53.6	35.0	16.1	53.6	35.0	16.1	53.6	35.0	16.1	31.5
Ethyl cellulose	4.2	4.2	4.2	4.2	4.2	4.2	4.2	4.2	4.2	4.2	4.2
Magnesium stearate	0.8	0.8	0.8	0.8	0.8	0.8	0.8	0.8	0.8	0.8	0.8

+ The quantity of raw materials is expressed in mg per tablet and

* DCPD is dicalcium phosphate dihydrate

**TABLE 3:** DESIGN LAYOUT FOR 3^2^ FACTORIAL DESIGN

Batch code	Real values		Transformed values		Dependent variables	
			
	X_1_	X_2_	X_1_	X_2_	Y_1_	Y_2_
VP1	1:1	1:2.5	-1	-1	33.14	5.84
VP2	1:1	1:5	-1	0	25.33	6.50
VP3	1:1	1:7.5	-1	1	23.10	6.72
VP4	1:2	1:2.5	0	-1	27.92	6.64
VP5	1:2	1:5	0	0	18.11	7.62
VP6	1:2	1:7.5	0	1	10.07	7.66
VP7	1:3	1:2.5	1	-1	25.36	6.98
VP8	1:3	1:5	1	0	14.38	7.68
VP9	1:3	1:7.5	1	1	7.50	7.32
VP10+	1:2.6	1:5.4	0.6	0.18	16.30	7.40

X_1_ is the ratio of drug to HPMC K4M in core tablets, X_2_ is the ratio of drug to HPMC K100M in coat, Y_1_ is the percentage drug release in first h, Y_2_ is the rate of drug release after first h and the check point batch is VP 10.^(+)^

### Evaluation of the tablets:

The angle of repose was measured using the fixed height funnel method^18^. Glass funnel was secured with its tip at a given height (H) above a graph paper placed on a horizontal surface. Granules were poured through the funnel until the apex of conical pile touched the tip of funnel. The angle of repose was calculated using formula: tan α = H/R, where α is the angle of repose and R is the radius of conical pile. The diameter, thickness and crushing strength of ten randomly selected tablets were determined using Dr. Scheleuniger tablet hardness tester (Pharmatron 8, Germany). Friability was evaluated as the percentage weight loss of twenty tablets tumbled in a friabilator (Electrolab, Model EF2, India) for 4 m at 25 rpm. The tablets were dedusted and the loss in weight caused by fracture or abrasion was recorded as the percentage friability.

The tablets were subjected to *in vitro* drug release for 12 h in a calibrated USP dissolution test apparatus (Electrolab, Model TDT 06-T, Mumbai) equipped with basket employing 900 ml phosphate buffer solution (pH 7.2). The baskets were rotated at 100 rpm and the dissolution medium was maintained at a temperature of 37±0.5^º^. Ten millilitre samples were withdrawn and analyzed spectrophotometrically at 225 nm using Shimadzu-1700 UV/Vis spectrophotometer after suitable dilution of the samples^19^,^20^. Equal volume of the fresh dissolution medium (10 ml, 37±0.5^º^ ) was replaced after each withdrawal. The absorbance values were transformed to concentration by reference to a standard calibration curve obtained experimentally (r^2^ = 0.999).

### Criteria for optimized batch:

Two limits were arbitrarily selected; i) y_1_: Percentage drug release in first h is equal to 16%, and ii) y_2_: drug release rate after first h is equal to 7.6% per h.

### Analysis of drug release data:

The method of Bamba *et al*. was adopted to ascertain kinetics of drug release^21^. *In vitro* drug release data were analyzed by fitting different kinetic models in order to evaluate the release mechanism of venlafaxine hydrochloride from the matrices (Table 4)^22^–^27^. A Fortran software, developed in-house, was used for computation of slope and intercept. The least value of sum of square of residuals (SSR) and Fisher’s ratio (F) were used to select the most appropriate kinetic model.

**TABLE 4:** MATHEMATICAL MODELS

Function	Equation
Zero-order	% diss* = kt
First-order	% diss* = 100 (1-e^-kt^)
Hixson-Crowell	% diss* = 100 [1-{1-(kt/4.6416)}^3^]
Higuchi	% diss* = kt^0.5^
Korysmeyer-Peppas	% diss* = kt^n^
Weibull	% diss* = 100 [1-e^-(t/Td)β^]

% diss* is the percent dissolved at time t, k is the dissolution rate constant, n is the release component, β is the shape parameter and Td is the time at which 63.2% of the drug is dissolved.

## RESULTS AND DISCUSSION

**Fig. 1:**
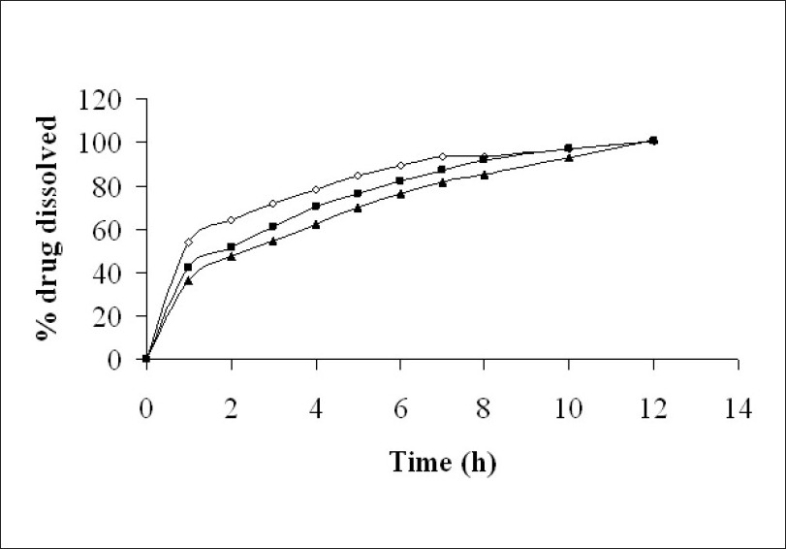
*In vitro* drug release from tablet of batch VH1-VH3. Release rate of formulated tablets (-◆-) Batch VH1, (-▲-) BatchVH2, (-▴-) Batch VH3.

For a freely soluble drug like venlafaxine hydrochloride (534 mg/ml in water), a rapid rate of hydration of matrixing agent is necessary. A slow polymer hydration rate may lead to dose dumping due to quick penetration of dissolution fluid in the tablet core. Hence, a rapidly hydrating hydrophilic matrixing agent (HPMC K4M) was selected. Granulation is the key process parameter in the production of controlled/delayed release dosage forms^28^. The physical properties of granules such as specific surface area, shape, hardness, surface characteristics and size can significantly affect the rate of drug dissolution^29^. The granules were evaluated for angle of repose (Table 1). A chapter on powder flow has been recently introduced in USP 29-NF 24^30^. The granules of batches VH1, VH2 and VH3 exhibited fair to passable flow. The diameter of formulated batches (VH1 to VH3) was 8±0.01 mm. The thickness of tablets of batches VH1 to VH3 increased from 2.0 to 2.7 mm due to increase in HPMC to drug ratio. The percentage friability for all the formulations (VH1 to VH3) was below 1%, indicating that the friability was within the prescribed limits^29^. Fig. 1 depicts that the tablets of preliminary batches (VH1 to VH3) exhibited burst release (>36% of drug in 1 h). The probable reason for faster drug release could be high aqueous solubility of the drug and short diffusion path. Hence, it was decided to coat the core tablet with HPMC K100M (batch VP0) using press coating technique.

**Fig. 2:**
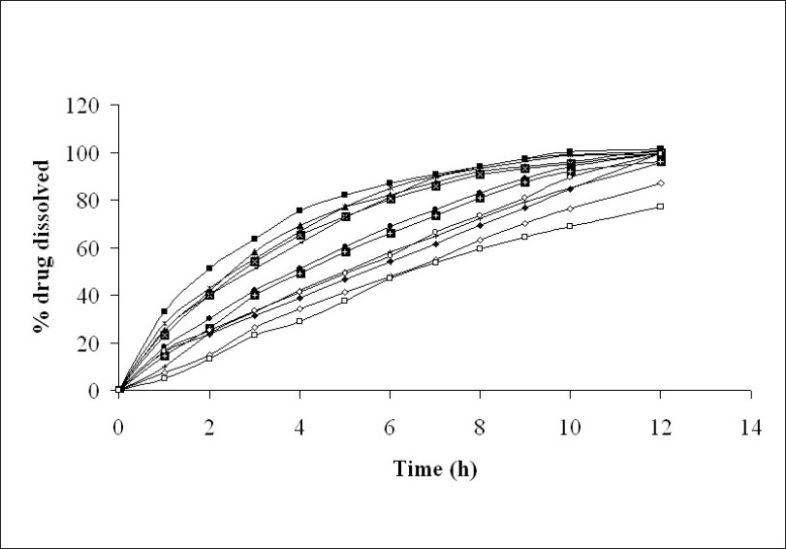
*In vitro* drug release from press coated tablets. Release rate of formulated tablets (—□—) Batch VP0, (—■—) Batch VP1, (—▲—) Batch VP2, (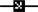) Batch VP3, (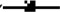) Batch VP4, (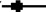) Batch VP5, (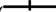) Batch VP6, (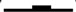) Batch VP7, (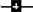) Batch VP8, (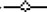) Batch VP9, (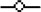) Batch VP10 and (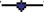) hypothetical drug release pattern

**Fig. 3:**
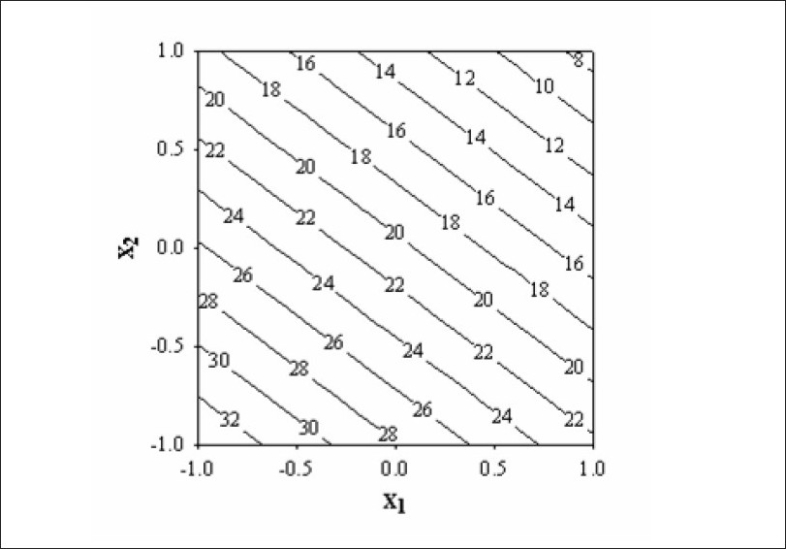
Contour plot showing amount of drug release at first h (y_1_) using different combination of x_1_ and x_2_. The contour lines shows percentage drug release at the end of first h.

The diameter and thickness of press coated tablets ranged from 10.29 to 10.31 and 3.93 to 3.97, respectively. The percentage friability of all formulated batches were within the limits (<1%). Fig. 2 reveals the amount of drug released from batches VP0 to VP10. The drug release from batch VP0 was noticeably low (5% in 1 h). Moreover, complete drug release was not observed in 12 h. To achieve the desired drug release (16% in 1h), twenty percentage drug was incorporated in the coat. A 3^2^ full factorial design was adopted to optimize the formulation. Batches VP5 and VP8 showed drug release close to the ideal requirement, i.e. 16% in first h. The slope (y_2_ ) of line correlating the amount of drug dissolved after first h versus time was computed to ascertain the drug release rate. The drug release rate from batch VP5, VP6, VP8 and VP9 was very close to that of the ideal value, i.e 7.6% per h. Further optimization was carried out by evolving mathematical models using multiple regression analysis. Eqn. 1 shows the relationship between the percentage drug released in first h (y_1_ ) and the independent variables. Fig. 3 represents contour plot for percentage drug release at first h (y_1_ ).:- y_1_ = 20.54-5.72x_1_-7.62x_2_--(1), (r^2^ = 0.94, *P*<0.05). Multiple regression analysis was adopted to evolve equation correlating the drug release rate after 1h (y_2_ ) with the independent variables (Eqn. 2). y_2_ = 7.57+0.48x_1_+0.37x_2_-0.4x_1_^2^-0.4x_2_^2^--(2), (r^2^ = 0.98, *P*< 0.05)

**Fig. 4:**
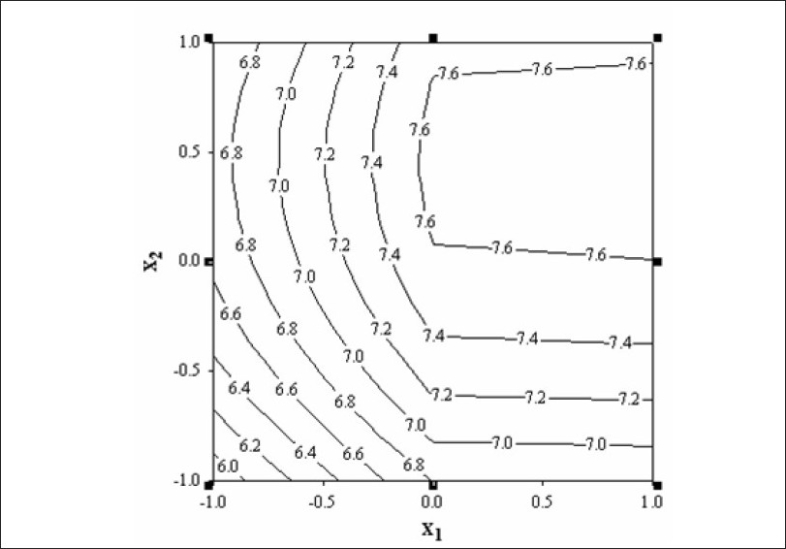
Contour plot showing amount of drug release every h for 11 h after first h (y_2_) using different combination of x_1_ and x_2_. The contour lines shows percentage drug release rate after first h.

Fig. 4 represents contour plot for drug release rate after first h. A check point batch (VP10) containing 1:2.6 and 1:5.4 drug to polymer in core and coat respectively was formulated. The computed and experimental values of y_1_ and y_2_ for batch VP10 were 15.8 and 7.75; 16.3 and 7.4 respectively. Similarity factor (f_2_ ) was calculated considering the ideal release profile as reference and batch VP10 as test formulation. The computed value of f_2_ was 73.6. Therefore, it was concluded that the two dissolution profiles were similar.

**Fig. 5:**
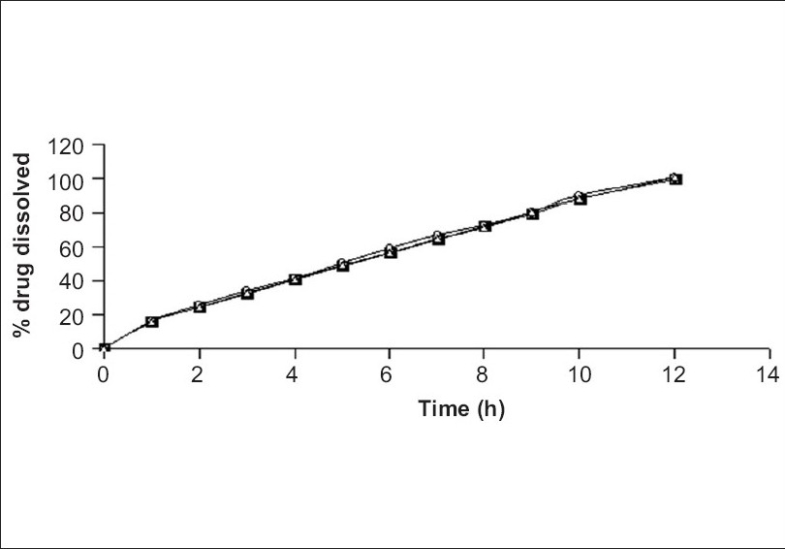
*In vitro* drug release from batch VP10 in different media. Release rate of formulated tablets (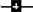) pH 7.2, (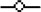) pH 5 and (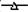) distilled water

The tablets of batch VP10 were investigated for *in vitro* drug release in three dissolution media (pH 5, 7.2 and distilled water). Fig. 5 reveals that tablets showed identical drug release in three dissolution media. The probable reason for getting the similar dissolution pattern in different dissolution media could be pH independent swelling and gelling of HPMC and similar drug solubility in the different dissolution media^31^.

The *in vitro* drug dissolution data of batch VP10 was analyzed for establishing kinetics of drug release. Model fitting was done using an in-house program developed by the authors. Zero-order, first-order, Higuchi, Hixson-Crowell, Korsmeyer-Peppas and Weibull models were tested (Table 4). Korsmeyer-Peppas model showed least sum of square of residuals (SSR = 75.8) and Fischer’s ratio (F=8.4). The mechanism of release of venlafaxine hydrochloride from the formulated batch was by anamolous non-fickian diffusion i.e. diffusion coupled with erosion.

## References

[1] Makhijal S, Vavia P (2002). Once daily sustained release tablets of venlafaxine: A novel antidepressant. Eur J Biopharm Pharm.

[2] Holiday S, Benfield P (1995). Venlafaxine: A review of its pharmacology and therapeutic potential in depression. Drugs.

[3] Muth E, Moyer J, Haskins J, Andree T, Husbands G (1991). Biochemical, neurophysiological and behavioral effects of Wy-45,233, its enantiomers and other identified metabolites of the antidepressant venlafaxine. Drug Dev Res.

[4] Parfitt K (1999). Martindale-The complete drug reference.

[5] Budavari S (1996). The Merk index-An encyclopedia of chemicals, drugs and biologicals.

[6] Mehta MC (1995). Physicians' desk reference.

[7] Sherman DM, Clark JC, Lamer JU, White SA (2004). Extended release formulation containing venlafaxine.

[8] Sherman DM, Clark JC, Lamer JU, White SA (2003). Extended release formulation containing venlafaxine.

[9] Sherman DM, Clark JC, Lamer JU, White SA (2001). Extended release formulation containing venlafaxine.

[10] Pisek R, Zupan CS, Sugula ZM (2006). Sustained release pharmaceutical composition comprising venlafaxine. PCT Int Appl WO 2006/010605 A2.

[11] Hiremath S, Saha R (2004). Design and study of rifampicin oral controlled release formulations. Drug Deliv.

[12] Sela Y (2003). Extended release compositions comprising as active compound venlafaxine hydrochloride. PCT Int Appl WO 03/041692.

[13] Molenda FA (2002). Process for preparation of programmed liberation composition with venlafaxine and the resulting product. PCT Int Appl WO 02/102129.

[14] Sela Y (2003). Extended release composition comprising as active compound venlafaxine hydrochloride.

[15] Seth P (2002). Tablet comprising a delayed release coating.

[16] Bhattacharya S, Pandita S, Mayank J, Kshirsagar R (2005). Extended release coated microtablets of venlafaxine hydrochloride.

[17] Vaya N, Karan RS, Madkarni SS, Gupta VK (2004). Novel drug delivery system.

[18] François, L, Louis C, Roch T (2002). New methods characterizing avalanche behavior to determine powder flow. Pharm Res.

[19] Dallet P, Labat L, Richard M, Langlois M, Dubost J (2002). A reversed-phase HPLC method development for the separation of new antidepressants. J Liq Chromatogr Relat Technol.

[20] Dallet PH, Labat L, Kummer E, Dubost J (2001). HPLC separation of new antidepressants on Satisfaction ® RP 18 AB and Stability ® C8 columns. Eur J Emerg Med.

[21] Bamba M, Puisievx F, Marty J, Carstensen J (1979). Release mechanism in gel forming sustained release formulation. Int J Pharm.

[22] Gibaldi M, Feldman S (1967). Establishment of sink conditions in dissolution rate determination. J Pharm Sci.

[23] Korsmeyer R, Gurny R, Doelker E, Buri P, Peppas N (1983). Mechanisms of solute release from porous hydrophilic polymers. Int J Pharm.

[24] Higuchi T (1961). Rate of release of medicaments from ointment bases containing drugs in suspensions. J Pharm Sci.

[25] Higuchi T (1963). Mechanism of sustained-action medication: Theoretical analysis of rate of release of solid drugs dispersed in solid matrices. J Pharm Sci.

[26] Higuchi I (1962). Analysis of data on the medicament release from ointments. J Pharm Sci.

[27] Langenbucher F (1972). Linearization of dissolution rate curves by the weibull distribution. J Pharm Pharmacol.

[28] Raghuram R, Srinivas M, Srinivas R (2003). Article 61 Once daily sustained release matrix tablets of Nicorandil: Formulation and *in vitro* evaluation. AAPS PharmSciTech.

[29] Banker GS, Anderson NR, Lachman L, Liberman HA, Kanig JL (1987). The Theory and Practice of Industrial Pharmacy.

[30] (2006). United States Pharmacopoeia XXIX, NF XIV. The United States Pharmacopoeial Convention Inc.

[31] Gohel M, Amin A, Patel K, Panchal M (2002). Studies in release behavior of diltiazem HCL from matrix tablets containing (hydroxypropyl) methyl cellulose and Xanthan gum. Boll Chim Farmac.

